# Sequencing and *De Novo* Assembly of the Gonadal Transcriptome of the Endangered Chinese Sturgeon (*Acipenser sinensis*)

**DOI:** 10.1371/journal.pone.0127332

**Published:** 2015-06-01

**Authors:** Huamei Yue, Chuangju Li, Hao Du, Shuhuan Zhang, Qiwei Wei

**Affiliations:** 1 Key Laboratory of Freshwater Biodiversity Conservation, Ministry of Agriculture of China, Yangtze River Fisheries Research Institute, Chinese Academy of Fisheries Science, Wuhan 430223, China; 2 Freshwater Fisheries Research Center, Chinese Academy of Fisheries Science, Wuxi 214081, China; Temasek Life Sciences Laboratory, SINGAPORE

## Abstract

**Background:**

The Chinese sturgeon (*Acipenser sinensis*) is endangered through anthropogenic activities including over-fishing, damming, shipping, and pollution. Controlled reproduction has been adopted and successfully conducted for conservation. However, little information is available on the reproductive regulation of the species. In this study, we conducted *de novo* transcriptome assembly of the gonad tissue to create a comprehensive dataset for *A*. *sinensis*.

**Results:**

The Illumina sequencing platform was adopted to obtain 47,333,701 and 47,229,705 high quality reads from testis and ovary cDNA libraries generated from three-year-old *A*. *sinensis*. We identified 86,027 unigenes of which 30,268 were annotated in the NCBI non-redundant protein database and 28,281 were annotated in the Swiss-prot database. Among the annotated unigenes, 26,152 and 7,734 unigenes, respectively, were assigned to gene ontology categories and clusters of orthologous groups. In addition, 12,557 unigenes were mapped to 231 pathways in the Kyoto Encyclopedia of Genes and Genomes Pathway database. A total of 1,896 unigenes, potentially differentially expressed between the two gonad types, were found, with 1,894 predicted to be up-regulated in ovary and only two in testis. Fifty-five potential gametogenesis-related genes were screened in the transcriptome and 34 genes with significant matches were found. Besides, more paralogs of 11 genes in three gene families (*sox*, *apolipoprotein* and *cyclin*) were found in *A*. *sinensis* compared to their orthologs in the diploid *Danio rerio*. In addition, 12,151 putative simple sequence repeats (SSRs) were detected.

**Conclusions:**

This study provides the first *de novo* transcriptome analysis currently available for *A*. *sinensis*. The transcriptomic data represents the fundamental resource for future research on the mechanism of early gametogenesis in sturgeons. The SSRs identified in this work will be valuable for assessment of genetic diversity of wild fish and genealogy management of cultured fish.

## Introduction

Sturgeons are one of the most primitive vertebrates with over 200 million years of history, leading to a vital evolutionary position. Among the different sturgeon species, different degrees of ploidy caused by genome duplication events were characterized [1, 2], which is another interesting field worth for exploration on sturgeons. Besides, as the source of caviar from the roe, the sturgeon is of high economic value [3], which becomes the primary factor leading to their exceedingly endangered status [4]. According to International Union for the Conservation of Nature (IUCN 2010) data, the sturgeon has been identified as one of the most endangered group of species, with 85% being on the edge of extinction.

The Chinese sturgeon (*Acipenser sinensis*), is an anadromous fish distributed in Yangtze River and East China Sea [5, 6]. It is currently endangered due to habitat degradation caused by anthropogenic activities such as damming, shipping, pollution and over-fishing [3, 7]. This is compounded by the difficulty in recovery of their numbers, considering the late sexual maturity (8–18 years for males and 14–26 years for females) as well as the reproduction interval of 2–7 years. Artificial propagation has been tried to conduct on the Chinese sturgeon since the later 1980s and has shown potential for species conservation. In 2012, gonadal maturation was reached in artificially bred Chinese sturgeons, from which 23,000 larvae were hatched by *in vitro* fertilization [8]. However, during rearing it is impossible to distinguish males from females morphologically. In addition the gonads of artificially bred Chinese sturgeon are smaller than those of the wild [8]. These factors constitute obstacles not only to the conservation of germplasm resources but also to the efficacy of artificial propagation. To resolve these issues, it is necessary to study the mechanisms of reproduction regulation and investigate genes involved in early gametogenesis.

Gametogenesis includes spermatogenesis and oogenesis. Spermatogenesis is composed of three phases: mitosis, meiosis and spermiogenesis. Spermatogenesis begins with the proliferation of spermatogonia which develop into primary spermatocytes [9]. After two meiotic divisions, the primary spermatocytes produce four spermatids, which do not divide further but undergo the spermiogenic process to transform into elongated spermatids [10]. The differentiation from spermatogonia to primary spermatocytes with the onset of meiosis is one of the most important steps in spermatogenesis, which involves the sex steroid hormones stimulation and the endocrine regulation [11, 12]. Despite this, many studies on spermatogenesis were primarily focused on the final maturation of male gametes [13, 14].

Oogenesis commences with the formation of primordial germ-cells (PGCs), which then transform into oogonia and develop further into primary oocytes [15]. The following oocyte development has been classified as the primary oocyte stage, cortical-alveolar stage, vitellogenic stage with the onset of meiosis, and the final maturation stage [16]. Oocyte growth during the early period of oogenesis is responsible for cytoplasmic organelles replication and redistribution, RNA synthesis, and nutrient incorporation. However, research on oogenesis was concentrated mainly on the late stages of vitellogenesis, final maturation and ovulation [17, 18], while stages of primary oocyte growth was mainly unexplored. In teleost fish, the early period of primary oocyte growth may occupy a long period of the lifespan in some species like sturgeons. For example, in female Chinese sturgeons of 5-year-old, the oocytes were still in the primary oocyte growth stage [19], and might last well over a decade at this stage during artificial breeding.

Transcriptome sequencing has been proven to be an effective means of gene discovery [4, 20], especially with the availability of high-throughput next generation sequencing technologies. Transcriptome sequencing is also emerging to be a rapid and efficient method of genetic marker development, including simple sequence repeats (SSRs) or microsatellites, which are useful for numerous studies like linkage mapping and genetic diversity research [21]. This study used Illumina sequencing to provide the first characterization of the Chinese sturgeon transcriptome. We performed transcriptome sequencing on the gonads of three-year-old Chinese sturgeon. The transcriptome dataset should be beneficial for differential gene expression between gonads during early gametogenesis. SSRs detected from the transcriptome data should also be helpful for conservation of this endangered species.

## Material and Methods

### Ethics statement

All fish handling and experimental procedures were approved by the Animal Care and Use Committee of the Yangtze River Fisheries Research Institute, Chinese Academy of Fishery Sciences.

### Experimental Fish and Sample Collection

One male and one female Chinese sturgeon, both 3-year-old immature individuals, were sampled at Taihu Station, Yangtze River Fisheries Research Institute, Chinese Academy of Fisheries Science. After anaesthetization by 0.05% MS-222 (Sigma, USA), testis and ovary tissues were collected and immediately immersed in the RNAlater (Ambion, Austin, TX, USA). Samples were stored at 4°C for 16 h, and then transferred to an ultralow freezer at -80°C until preparation of RNA. Small pieces of gonad were fixed in Bouin’s solution and embedded in paraffin. They were cut at 8 μm and stained with hematoxylin and eosin (HE).

### RNA Isolation, Library Construction and Illumina Sequencing

Isolation of total RNAs was carried out by RNeasy Plus Mini Kit (Qiagen, Dusseldorf, Germany) according to the manufacturer’s instructions. The RNA integrity and concentration was determined by Nanodrop-2000 spectrophotometer (Thermo, USA) and Agilent 2100 Bioanalyzer (Agilent, Santa Clara, CA). Genomic DNA was removed by RNase-free DNase I (Qiagen) for 30 min at 37°C. The mRNA-seq library was constructed with the mRNA-seq Sample Preparation Kit (Illumina, San Diego, CA) as described [22]. Briefly, enriched mRNA was broken into short fragments, which were used for the first-strand cDNA synthesis, followed by the second-strand cDNA synthesis. The double-strand cDNA was then end-paired, tailed and ligated with the PE Adapter Oligo Mix (Illumina). Final cDNA libraries were obtained by PCR amplification and purification, and were paired-end sequenced with the Illumina HiSeq 2000 (Biomarker Technologies Co., Ltd, Beijing, China).

### *De novo* assembly of sequencing reads

Raw reads (2*101 bp paired-end sequencing) of the two transcriptome datasets (testis and ovary) were cleaned by filtering out adaptor-only and low quality reads. The adaptor-only reads (nt length of recognized adaptor ≤13 and the remaining adaptor-excluded nt length ≤35) were filtered out by the software WipeAadpter.pl (Biomarker Technologies Co., Ltd, Beijing, China). Reads with more than 50% of bases having a Q-value ≤20 were filtered out by the software Fastq_filter (Biomarker Technologies Co., Ltd, Beijing, China). Clean reads were then assembled with the short reads assembling program Trinity, version Trinityrnaseq_r2012-06-08 [23], with the parameters set at a similarity of 90%. Trinity contains three software modules: Inchworm, Chrysalis and Butterfly. In the process of Inchworm, overlapping k-mers (in this study, k = 25), which forms a k-mer dictionary, are generated from decomposition of each sequence read. Then the likely error-containing k-mers were removed from the library, and the k-mer most frequently appeared in the dictionary will work as the seed of the Inchworm contig. For each of the k-mer seeds, another k-mer, whose sequence could match the seed by k-1 times in either end and occurs most frequently in the dictionary, is anchored to extend the seed on the terminal base. If the neighbor k-mer is detected in neither direction, the growing of sequence terminates to produce an Inchworm contig. In the second phase of trinity, Chrysalis, the abundant contigs produced by the repeated Inchworm processes are built into de Bruijn graphs through k-1 overlaps. In the last step, Butterfly, the fragmented de Bruijn graphs are trimmed, compacted and reconciled to final linear transcripts [23]. Redundant sequences were eliminated, and the longest transcripts were recognized as unigenes, which were grouped together to conduct the final assembly for the following annotation.

### Transcriptome annotation and ontology

Open reading frames (ORFs) of transcript and unigene sequences were predicted by the TransDecoder package (http://transdecoder.sourceforge.net/), with the minimum ORF length of 100 bp. Unigene sequences were assigned to the NCBI Nt database (downloaded in August, 2013) (BLASTn), the NCBI non-redundant (Nr) protein databases (downloaded in August, 2013), the Swiss-Prot protein database and the Translated EMBL Nucleotide Sequence Database (TrEMBL) (downloaded in August, 2013) (BLASTx) with an E-value of 1e-5. Each unigene sequence was allocated a gene name by BLAST hit with the highest score. The ‘‘getorf” program of EMBOSS software package (version 1.20.0) [24] was employed to predict the Open reading frames (ORFs), and the longest ORF was selected for each unigene by defaulting parameters.

Homology searches were performed by comparison against the NCBI Nr protein database with the Blastx algorithm (E-value cutoff of 1e-5) [23]. With Nr annotation, the Blast2GO program [24] was employed to produce Gene Ontology (GO) (database downloaded in March 2013) annotations. Functions of the sequences were also predicted by query of the Clusters of Orthologous Groups (COG) database (http://www.ncbi.nlm.nih.gov/COG/, database downloaded in April 2013) (BLASTx, E-value cutoff of 1e-5). The assembled sequences were assigned to the Kyoto Encyclopedia of Genes and Genomes (KEGG) pathways (database downloaded in October 2011) using the online KEGG Automatic Annotation Server (KAAS) (http://www.genome.jp/kegg/kaas/) (BLASTx, E-value cutoff of 1e-5). KEGG Orthology (KO) assignment was obtained with the bi-directional best hit (BBH) method [25]. KO assignments and KEGG pathways that are fulfilled with the KO assignments were combined to constitute the final output of KEGG analysis.

### Digital gene expression analysis

Numbers of reads in the RNA-Seq analyses were normalized with reads per kilo base of transcripts per million (RPKM) to compute the gene expression levels [26]. Detection of differentially expressed genes were performed by EBseq software (version 1.1.7) [27] in pair-wise comparison. The Benjamini–Hochberg false discovery rate (FDR < 0.01) was adopted for multiple testing correction of the result. Genes were defined as differentially expressed by parameters of FDR <0.001 and the absolute value of the log2 ratio >1 (The RPKM values of the gene in one sample was at least two fold that in another sample).

### Microsatellite markers

Unigenes of length >1,000 bp were submitted to the software MISA (MIcroSAtellite; http://pgrc.ipk-gatersleben.de/misa, version 1.0) for SSRs detection. Six types of SSRs were examined: mono-, di-, tri-, tetra-, penta- and hexanucleotide repeats, and the compound SSR [the sequence containing more than one type of repeat units, e.g., (GT)n(AT)m]. Standards of the repeat unit numbers were set as follows: mono-10, dimer-6, trimer-5, tetramer-5, pentamer-5, and hexamer-5.

### Relative real-time PCR validation

To evaluate the validity of Illumina analysis and assess the expression profiles with respect to specific mRNA abundances, 10 putative genes were chosen and detected by qRT-PCR. The cDNA templates used were reverse-transcribed with PrimeScript RT reagent Kit (Takara, Japan) as instructed. Relative real-time PCR was performed in a volume of 20 μL with a dsDNA-binding dye, SYBR green real-time PCR master mix (BioRad) on a DNA Engine Chromo 4 real-time system (BioRad) as described [28]. The primers used are listed in S6 Table. Each sample was analyzed in triplicates. The relative expression level of target genes was measured with the 2^−ΔΔCT^ method and normalized by the median expression of *β-actin*. The data was presented as mean ± SD. The difference was considered statistically significant at *P*-value < 0.05 assessed by the Student's t-test.

## Results

### Sequence analysis and assembly

**Fig 1:**
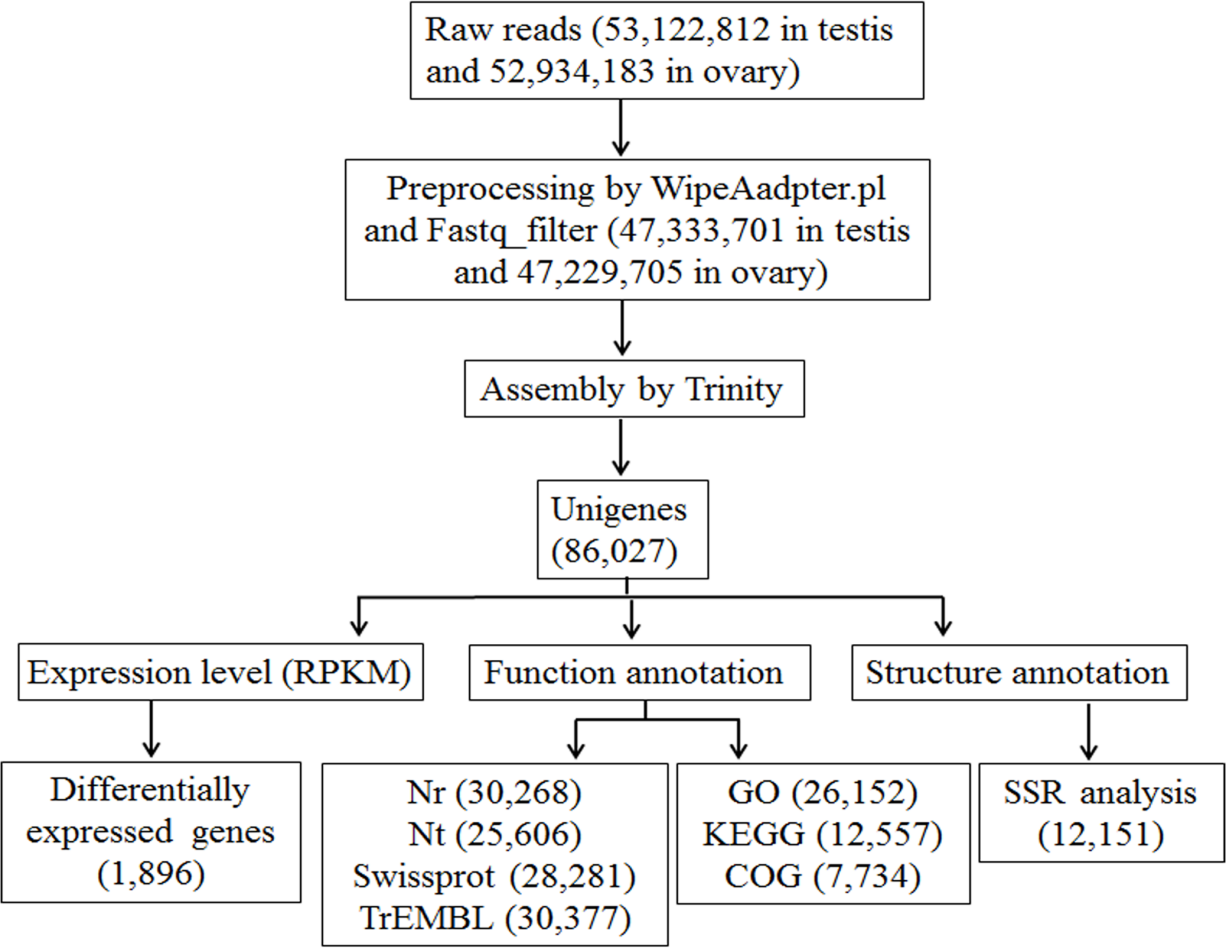
Transcriptome assembly and analysis pipeline. Clean reads were obtained by pre-processing the raw reads with WipeAadpter.pl software and Fastq_filter software. A total of 86,027 unigenes were acquired by Trinity assembly. The expression levels of the unigenes were normalized with RPKM, through which 1,896 genes differentially expressed between ovary and testis were identified. The unigenes were used for functional annotation and SSR detection.

To obtain gonad transcriptome information of the Chinese sturgeon, cDNA libraries were constructed from the testis of an immature male sturgeon and the ovary of an immature female sturgeon, and sequencing runs were performed on the Illumina HiSeq 2000. The testis was composed mainly of the spermatogonia and primary spermatocytes, and the ovary was full of pre-vitellogenic oocytes (S1 Fig). The analysis pipeline began with preprocessing and ended with functional annotation and genetic markers (Fig 1). After stringent quality assessment and data filtering, 47,333,701 and 47,229,705 high quality reads from the testis and ovary, respectively, were selected for further analysis. The GC percentages were 51.73% (with 100% Q20 bases) from testis and 44.89% (with 100% Q20 bases) from ovary (Table 1). All sequence reads generated have been submitted to the NCBI Genbank under accession number SRP035284.

**Table 1:** Assembly statistics of the gonad transcriptome using the Trinity software package.

Total number of clean reads in testis library	47,333,701
Total number of clean reads in ovary library	47,229,705
GC percentage in testis library (%)	51.73
GC percentage in ovary library (%)	44.89
Total number of transcripts^a^	176,434
Mean length of transcripts (bp)	950
N50 length of transcripts (bp)	1,621
Total number of unigenes^b^	86,027
Mean length of unigenes (bp)	706
N50 length of unigenes (bp)	1,221

^a^ Sequences constructed from Inchworm contigs by Chrysalis module and Butterfly module.

^b^ The longest transcripts in the cluster units.

The Trinity program was used for the assembly of the RNA-Seq short reads, and a total of 12,802,085 contigs were assembled with the N50 length of 47 bp and the mean length of 45.25 bp (Table 1). A total of 176,434 transcripts were gained with the N50 length of 1,621 bp and an avarage length of 950 bp. The N50 length and mean length of the 86,027 unigenes produced was 1,221 bp and 706 bp, respectively (Table 1). Additionally, the transcript and unigene sequences were assigned with an ORF predictor TransDecoder, from which 42,227 (23.96%) transcripts (S1 Table) and 13,187 (15.33%) unigenes (S2 Table) were determined to contain complete ORFs.

### Sequence annotation

BlastX was used to search various protein databases, and the Nr, Nt, Swiss-Prot, KEGG, COG, GO and TrEMBL databases were employed for annotation of the 36,157 unigene sequences (Table 2). Significant matches were found for 12,897 (83.71%) unigenes (≥1000 bp) in the Nr database, 11,809 (70.81%) in the Nt database, and 12,604 (78.81%) in the Swiss-Prot database. In the KEGG, COG, GO, and TrEMBL databases, there were 5,943 (34.72%), 4,479 (21.39%), 11,962 (72.33%), and 12,885 (84.01%) significantly matched unigenes (≥1000 bp), respectively. The Nr database queries revealed *A*. *sinensis* sequences to closely match sequences of *Danio rerio* (17.30%), *Maylandia zebra* (10.10%), *Oreochromis niloticus* (7.82%), *Xenopus (Silurana) tropicalis* (7.31%), and *Chelonia mydas* (6.93%).

**Table 2:** Functional annotation of unigenes of the *Acipenser sinensis* transcriptome.

Databases	All Annotated transcripts	≤300 (bp)	300–1000 (bp)	≥1000 (bp)
nr	30,268	5,550	11,821	12,897
nt	25,606	4,147	9,650	11,809
Swissprot	28,281	4,865	10,812	12,604
KEGG	12,557	2,127	4,487	5,943
COG	7,734	944	2,311	4,479
GO	26,152	4,412	9,778	11,962
TrEMBL	30,377	5,631	11,861	12,885
All_Annotated	36,157	7,404	14,873	13,880

**Fig 2:**
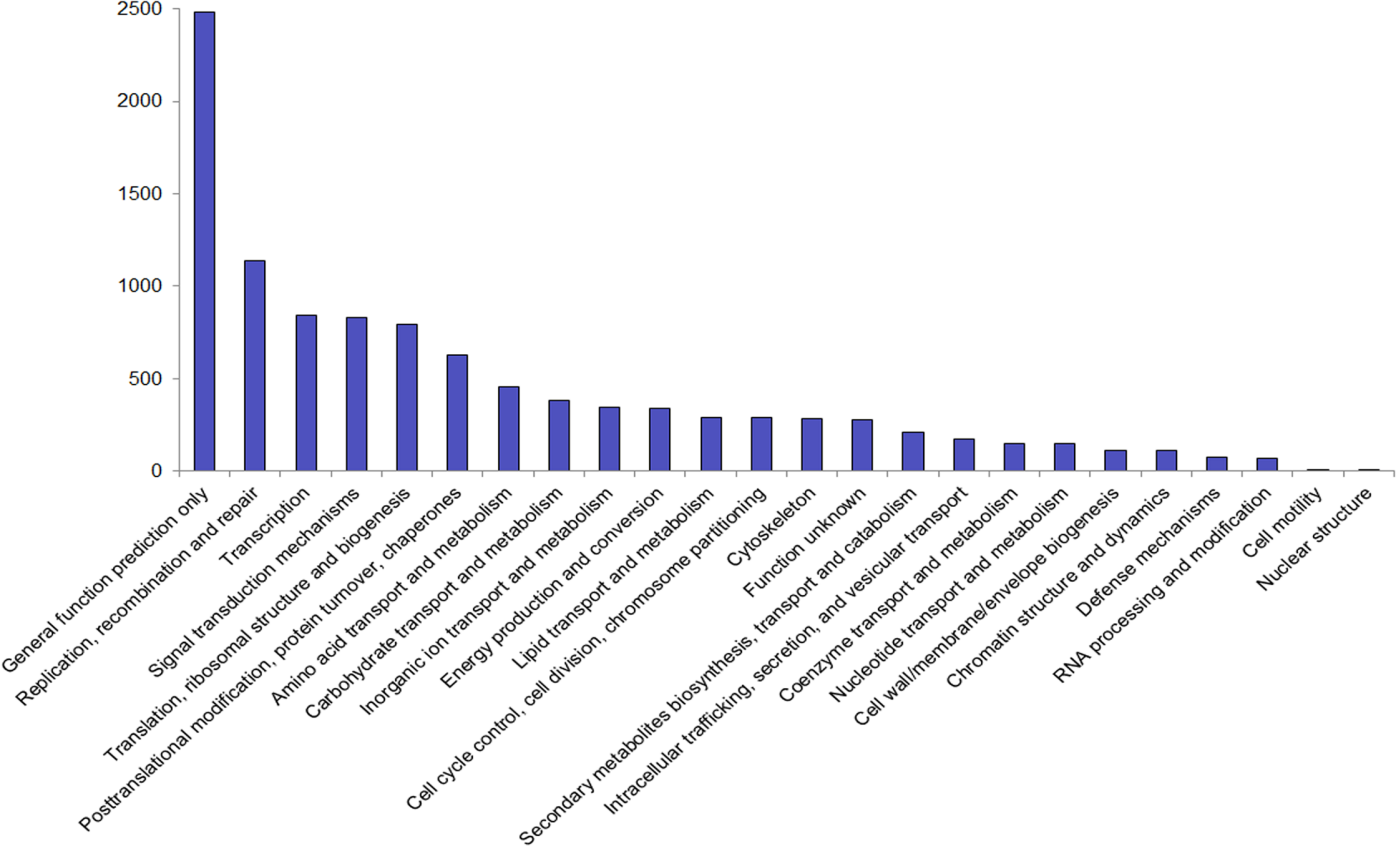
Clusters of orthologous groups (COG) classification. 4,479 unigenes with Nr hits were grouped into 24 COG classifications.

Functional prediction and classification of the unigene sequences were achieved by search against the COG database and a classification of 24 categories was obtained accordingly (Fig 2). The cluster for *‘general function prediction only’* constituted the major group (2,483; 23.79%), followed by *‘replication*, *recombination*, *and repair’* (1,135; 10.87%); *‘transcription’* (846; 8.10%); *‘signal transduction mechanisms’* (828; 7.93%); and *‘translation*, *ribosomal structure*, *and biogenesis’* (795; 7.62%). Only nine unigenes were allocated to *‘cell mobility’* and two to *‘nuclear structure’*. No unigenes were found to be related to *‘extracellular structures’* (Fig 2).

**Fig 3:**
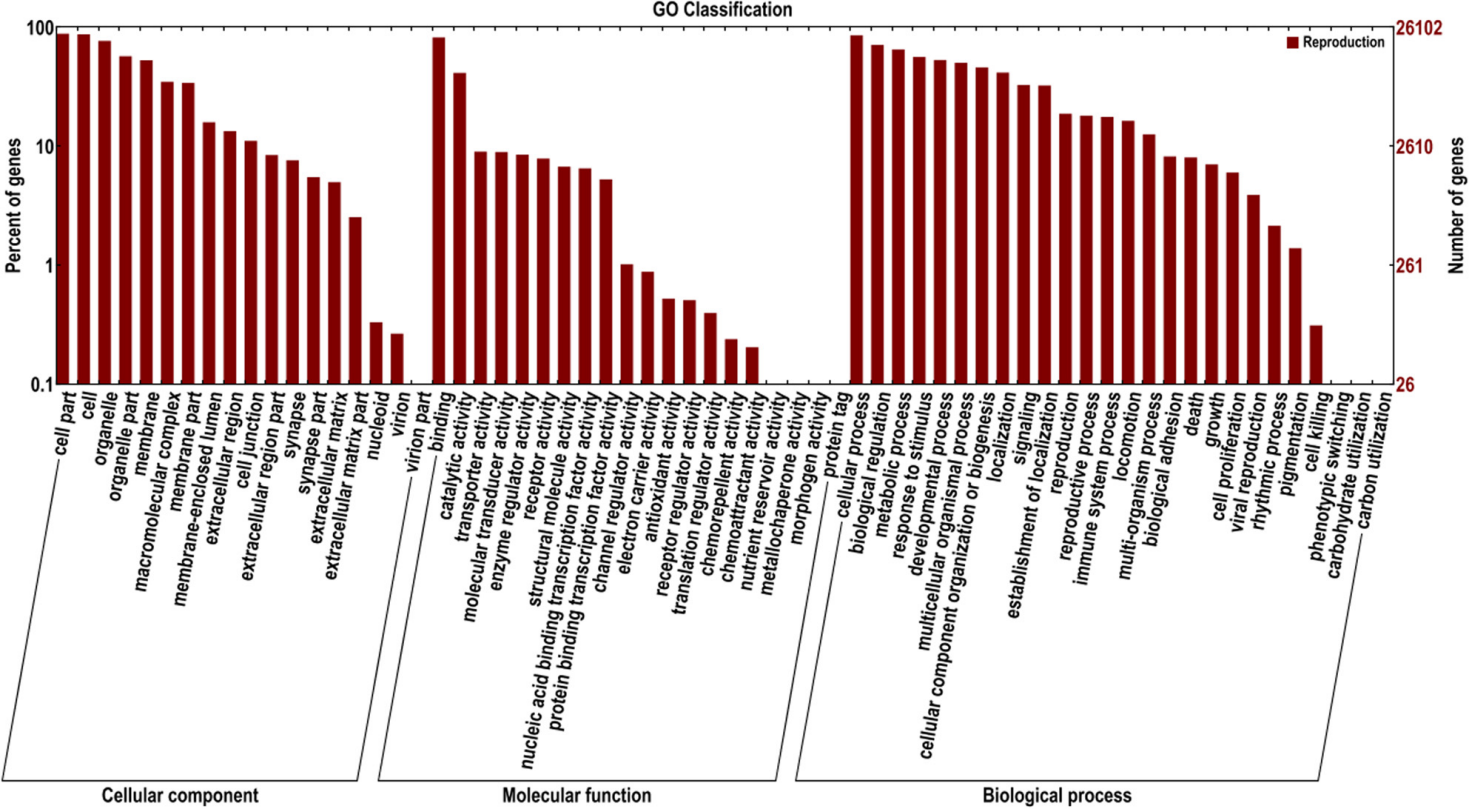
Functional annotation of assembled sequences based on gene ontology (GO) categorization. GO analysis was performed for three main categories: cellular components, molecular function, and biological processes.

In the GO annotation, the 26,152 unigenes were allocated one or more GO terms based on sequence similarity. The three main categories of GO annotations were 130,517 (37.50%) for cellular components, 46,967 (13.50%) for molecular function, and 170,512 (50%) for biological processes (Fig 3). For cellular components, genes involved in *‘cell part’* and *‘cell’* terms were the most represented. In the category of molecular function, the term *‘binding’* was in the highest proportion of annotations, followed by *‘catalytic activity’*. For biological processes, the most frequent GO term was *‘cellular process’*.

To further demonstrate the biological pathways involved in *A*. *sinensis*, the unigene sequences were mapped to the KEGG Pathway Tools. This process assigned 12,557 unigenes to a total of 231 pathways (S3 Table). These predicted pathways contained most biological pathways involved in the process of reproduction, including *GnRH* signaling (160 unigenes), steroid hormone biosynthesis (23), oocyte meiosis (170), regulation of actin cytoskeleton (341), DNA replication (48), RNA polymerase (32), mismatch repair (26), purine metabolism (245), adherens junction (155), cell cycle (215), Fc gamma R-mediated phagocytosis (52), and pyrimidine metabolism (123). Further information on these predicted pathways may be useful for investigations of their functions in *A*. *sinensis*.

### Search for genes involved in gametogenesis

**Fig 4:**
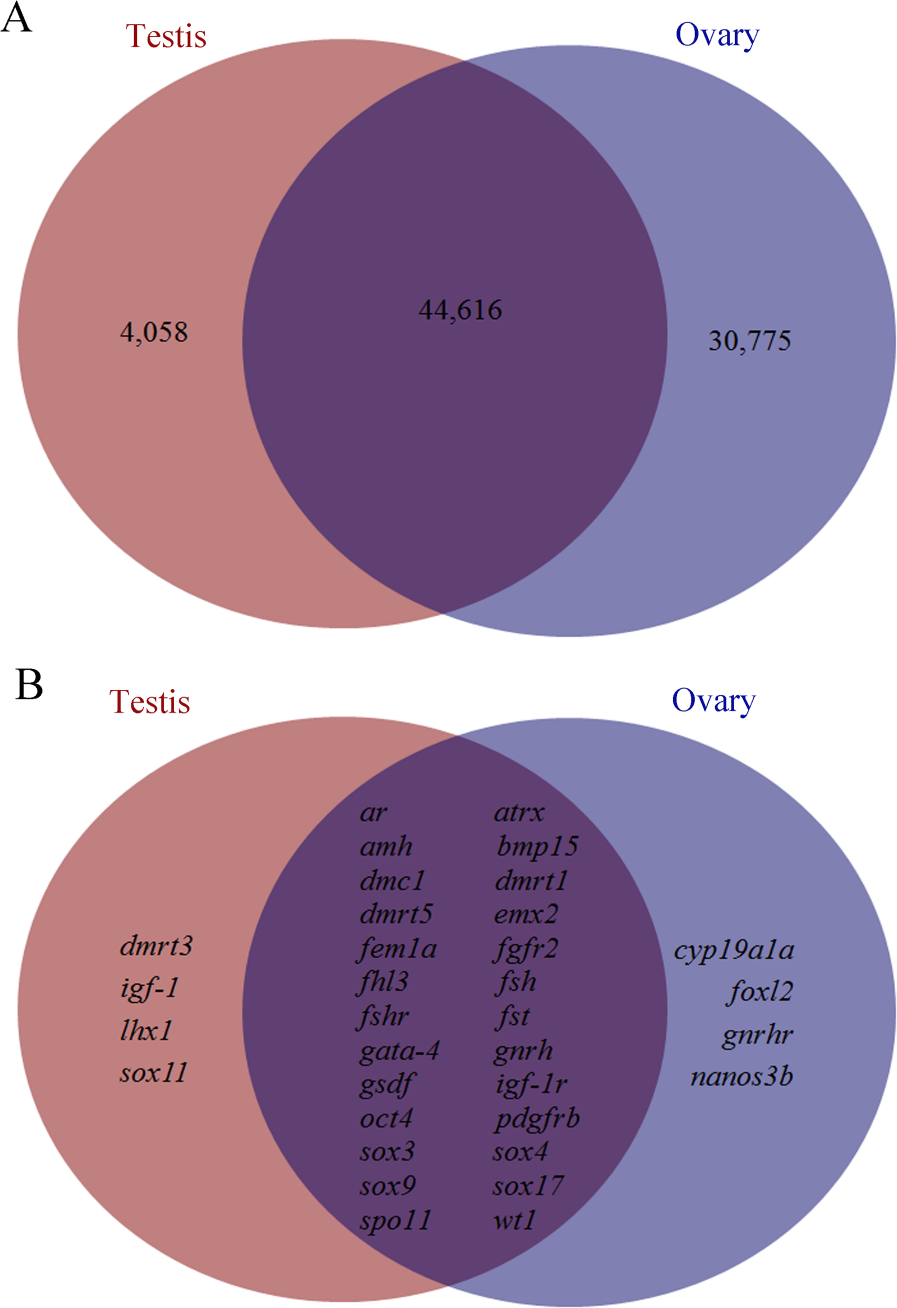
The distribution of transcripts between testis and ovary. (A) The numbers of all transcripts present in the testis, ovary, or both. (B) The distribution of transcripts with potential function in sexual development in testis and ovary.

We chose 55 genes reported as active in sexual development [4, 29] to search for unigenes annotated in the transcriptome of *A*. *sinensis* (S4 Table). There were significant matches with 34 of the 55 genes. Two major sex-determining genes of fish, *dmrt1* and *gsdf*, were also present in the gonad transcriptome of Chinese sturgeon (S4 Table). Thirty-two additional genes (S4 Table) involved in sexual development were investigated: five genes belonging to the *sox* subfamily, *sox3*, *sox4*, *sox 9*, *sox 11* and *sox 17*; 11 transcription factor genes, *bmp15*, *emx2*, *fem1a*, *fhl3*, *foxl2*, *gata4*, *lhx1*, *nanos3b*, *oct4*, *spo11* and *wt1*; seven receptors *ar*, *atrx*, *fgfr2*, *fshr*, *gnrhr*, *igf-1r* and *pdgfrb*; two hormone genes, *gnrh* and *fsh*; two genes belonging to the double sex and mab-3 (DM) domain, *dmrt3* and *dmrt5*; the signaling molecule *igf-1*; the recombinase *dmc1*; and the steroidogenic enzymes *amh*, *fst* and *cyp19a1a*. The expression levels (RPKM) of these transcripts are listed in S4 Table. Among these transcripts, *dmrt3*, *igf-1*, *lhx1*, and *sox11* were found to be present only in the testis transcriptome, while *cyp19a1a*, *foxl2*, *gnrhr* and *nanos3b* were present only in the ovary transcriptome (Fig 4B). The remaining genes were transcribed in both gonads. A total of 4,058 unigenes were transcribed exclusively in testis (RPKM>0), and 30,775 unigenes were found only in ovary, with 44,616 unigenes present in both testis and ovary (Fig 4A).

### Differentially expressed genes in early gametogenesis and confirmation using relative real-time PCR

EBseq software was used for analysis of genes differentially regulated in testis and ovary. Since biological replicates were unavailable, the following data are qualitative indications with no statistically significant differences. It was shown that 1,894 unigenes were up-regulated in ovary, while only two unigenes were up-regulated in testis (S5 Table). The most predominant up-regulated unigenes in the ovary were the zona pellucida (*ZP*) genes. In testis, one of the up-regulated unigenes (Reproduction_Unigene_BMK.74160) was the *Vitrin-like* gene, while the other up-regulated unigene (Reproduction_Unigene_BMK.66097) had no Nr annotation. No differential expression in testis and ovary was found for the sex-determining genes *dmrt1* and *gsdf*.

Ten potential genes involved in reproduction regulation were chosen for validation using real-time PCR. Nine (*zp3*, *zpax*, spermidine synthase, hatching enzyme, *pou2*, *Bucky ball*, *cyclin E*, *sox3*, and *gdf9*) were found to be up-regulated in ovary compared to testis, with the *Vitrin-like* gene showing higher expression in testis than in ovary, in accordance with the transcriptome analysis (Table 3). Previous RT-PCR detection of the *pou2* gene also suggested its higher transcription level in ovary [30]. These selected genes showed significant expression differences in ovary and testis, but at different levels from those found in transcriptome sequencing (Table 3).

**Table 3:** Real-time PCR confirmation of the relative expression of genes showing differential expression between the two gonad types.

Unigene ID	Illumina (O/T)^a^	qRT-PCR (O/T)^b^	Nr Annotation
75530	14.44	29.00	zona pellucida glycoprotein 3
59852	13.45	16.50	zona pellucida glycoprotein AX
57743	7.22	7.69	spermidine synthase
54618	9.58	20.00	hatching enzyme
73936	7.61	12.50	Pou2
75228	5.59	11.11	bucky ball
71078	5.11	20.00	E-type cyclin
60411	4.42	16.67	SRY (sex determining region Y)-box 3
74439	3.90	12.50	growth/differentiation factor 9
74160	-7.47	-10.90	predicted: vitrin-like

^a^ The relative-fold expression determined by Illumina transcriptome analysis.

^b^ The relative-fold expression validated by qRT-PCR.

O/T: The relative transcription level of genes in ovary compared to that in testis. O, Ovary; T, Testis.

### Transcriptome comparison of *A*. *sinensis*, *A*. *naccarii*, and *A*. *fulvescens*

We compared the *A*. *sinensis* transcriptome data with the already published transcriptome results of *A*. *naccarii* and *A*. *fulvescens* (Table 4). For sequencing of *A*. *naccarii* and *A*. *fulvescens*, Roche’s 454 platform was used, while Illumina Hiseq 2000 platform was employed for *A*. *sinensis*. In sequencing of the three species, tissues of male and female gonads were selected, with brain tissues included in *A*. *naccarii*. In *A*. *sinensis* and *A*. *naccarii*, 12,802,085 and 42,193 contigs were assembled, respectively. In *A*. *fulvescens*, the male assembly produced 12,791 contigs, while the female assembly (the ovary library and the female gonad with heterogametic sex library) produced 32,629 contigs.

**Table 4:** Transcriptome comparison of three sturgeon species.

Species	Platform	Tissue source	Clean reads	Contig	Singleton	Unigene
*A*. *sinensis*	Illumina	Testis	47,333,701	12,802,085		86,027
		Ovary	47,229,705			
*A*. *naccarii*	454	Testis+ brain	153,215	42,193	13,089	
		Ovary+brain	175,198			
*A*.*fulvescens*	454	Testis	134,278	12,791	99,713	
		Ovary	69,366	32,629^b^	154,724	
		Heterogametic^a^	269,913			

^a^ Female gonad with heterogametic sex

^b^ Contigs for female assembly with combined reads of the ovary and the female gonad with heterogametic sex

### Ortholog comparison between *A*. *sinensis* and *Danio rerio*

*Acipenser sinensis* is a functional tetraploid species [1], therefore more paralogs should be present compared to diploid teleosts. Here we searched the orthologs of 11 genes in three gene families (*sox*, *apolipoprotein* and *cyclin*) in *A*. *sinensis* and in *D*. *rerio*, a well-studied diploid teleost model. Of the *sox* family, additional paralogs of *sox3*, *sox6*, and *sox11*, were found in *A*. *sinensis* not present in their orthologs in *D*. *rerio* (Table 5). In the *apolipoprotein* superfamily, three more paralogs of apolipoprotein A-I (*apoA-I*) and two more of apolipoprotein E (*apoE*), together with an additional apolipoprotein L (*apoL*) paralog, were found in *A*. *sinensis*. Compared to *D*. *rerio*, an additional *cyclin A1*, *cyclin A2*, and *cyclin B3* paralog of the *cyclin* family was found in *A*. *sinensis*. Two more paralogs of *cyclin B1* and cyclin-dependent kinase 12 (*cdk12*) of the *cyclin* family were present in *A*. *sinensis* (Table 5).

**Table 5:** Comparison of orthologs from three gene families in Chinese sturgeon and zebrafish.

	*Acipenser sinensis*		*Danio rerio*	
Paralog Name	Numbers	Annotation Number	Numbers	Genbank Number
*sox3*	2	Unigene_BMK.60411	1	BAD11369
		Unigene_BMK.60413		
*sox6*	3	Unigene_BMK.66469	2	AEO16862
		Unigene_BMK.66470		AEO16863
		Unigene_BMK.66471		
*sox11*	3	Unigene_BMK.55711	2	AAI15165
		Unigene_BMK.83418		NP_571412
		Unigene_BMK.6537		
*apoA-I*	4	Unigene_BMK.41555	1	NP_571203
		Unigene_BMK.48306		
		Unigene_BMK.81617		
		Unigene_BMK.9655		
*apoE*	4	Unigene_BMK.11762	2	XP_005173815
		Unigene_BMK.4517		AAH65592
		Unigene_BMK.64153		
		Unigene_BMK.1736		
*apoL*	4	Unigene_BMK.43849	3	XP_005171358
		Unigene_BMK.60451		XP_005171359
		Unigene_BMK.18017		XP_005171360
		Unigene_BMK.65059		
*cyclin A1*	2	Unigene_BMK.40637	1	NP_997983
		Unigene_BMK.73469		
*cyclin A2*	2	Unigene_BMK.55138	1	NP_694481
		Unigene_BMK.60588		
*cyclin B1*	3	Unigene_BMK.63792	1	NP_571588
		Unigene_BMK.47153		
		Unigene_BMK.71665		
*cyclin B3*	2	Unigene_BMK.73792	1	NP_001070187
		Unigene_BMK.62679		
*cdk12*	3	Unigene_BMK.79801	1	XP_003200579
		Unigene_BMK.16306		
		Unigene_BMK.66799		

### Discovery of microsatellites

To explore the SSR profile in unigenes of Chinese sturgeon, the 16,687 unigene sequences were analyzed by MISA software. In the 7,654 unigene sequences investigated, 12,151 SSRs were detected, with 2,886 (37.71%) sequences containing more than one SSR. Five types of SSRs were found, among which the mono-nucleotide repeat motif represented the largest group (65.39%), followed by the dimer (18.71%), trimer (14.14%), tetramer (1.74%), and pentamer repeat motifs (0.02%) (Table 6).

**Table 6:** Summary of simple sequence repeat (SSR) types in the Chinese sturgeon transcriptome.

Repeat motif	Number^a^	%^b^
**Mono-nucleotide**		
A/T/C/G	**7,946**	**65.39**
**Di-nucleotide**		
AC/GT/AG/CT	1,548	
AT/AT	724	
CG/CG	1	
**Di-nucleotide Total**	**2,273**	**18.71**
**Tri-nucleotide**		
AAC/GTT/AAG/CTT/ AAT/ATT	622	
ACC/GGT/ACG/CGT/ACT/AGT	76	
AGC/CTG/AGG/CCT/ATC/ATG	1,006	
CCG/CGG	14	
**Tri-nucleotide Total**	**1,718**	**14.14**
**Tetra-nucleotide**		
AAAC/GTTT/AAAG/CTTT/ AAAT/ATTT	105	
AACC/GGTT/AACT/AGTT/	4	
AAGC/CTTG/AAGG/CCTT	7	
AATC/ATTG/AATG/ATTC/AATT/AATT	19	
ACAG/CTGT/ACAT/ATGT ACCT/AGGT	34	
ACGC/CGTG/ACGG/CCGT	4	
ACTC/AGTG/ACTG/AGTC	7	
AGAT/ATCT/AGCC/CTGG	13	
AGGC/CCTG/AGGG/CCCT	13	
ATCC/ATGG	5	
**Tetra-nucleotide Total**	**211**	**1.74**
**Penta-nucleotide**		
ACAGT/ACTGT	1	
ACCTG/AGGTC	1	
ATCCC/ATGGG	1	
**Penta-nucleotide Total**	3	**0.02**
**Grand Total**	**12,151**	**100.00**

^a^Number of SSRs detected in unigenes

^b^Relative percent of SSRs with different repeat motifs among the total SSRs

## Discussion

High throughput transcriptome sequencing has been widely used in numerous studies such as gene expression profiling, and simultaneous identification of mutations, sequence aberrations, and alternative splice variants [31]. Our study provides the first attempt into the investigation of transcriptome of the Chinese sturgeon. In total, 86,027 unigenes with the mean length of 705.84 bp were assembled. The unigenes obtained were further annotated with various protein databases, and were used to detect SSRs. These results should be meaningful for further investigation on early gametogenesis and conservation of the Chinese sturgeon.

In teleosts, as in other vertebrates, the regulation of reproductive process relies mainly on the neuroendocrine system, the brain–pituitary–gonad (BPG) axis [32]. In this axis, gonadotropin-releasing hormone (GnRH) promotes the synthesis and release of two specific pituitary hormones, most dominantly follicle stimulating hormone (FSH) and luteinizing hormone (LH). FSH and LH then activate receptors and stimulate synthesis of the various sex steroid hormones in the gonads to regulate steroidogenesis and gametogenesis [33, 34]. In the KEGG pathway annotation of Chinese sturgeon transcriptome in this study, a number of unigenes was assigned to the GnRH pathway (S3 Table), which was in accordance with its regulatory role during the gametogenesis in Chinese sturgeon. Besides, *sbgnrh*, *gnrhr*, *fsh* and *fshr* transcripts, except for *lh* and *lhr* (*lh* receptor), were found in the Chinese sturgeon transcriptome (Fig 4 and S4 Table). In our previous study, one GnRH precursor *AsGnRH1* transcript (the original Nr annotation for *sbgnrh* found in this study) was found in the testis and ovary of 4-year-old males and females [28], which was consistent with the detection of this study. In another research on the expression pattern of FSHβ and LHβ revealed the presence of both FSHβ and LHβ in mature Chinese sturgeon; while in the immature 4-year-old male Chinese sturgeon, only FSHβ was detected [35]. It might be that LHβ will only be expressed in the maturation period, but not in the gametogenesis period, just like the expression pattern of LH reported in salmonids [36–38].

The regulation of reproduction is a complex process that requires not only the control of HPG axis but also the cooperation of many autocrine and paracrine factors, including the insulin-like growth factor 1 (Igf-1). By binding to their receptors, Igf-1 plays very important role in spermatogenesis, Leydig cell differentiation and proliferation [39, 40]. In the Chinese sturgeon transcriptome, we found *igf-1* transcript transcribed only in the testis with no transcription in the ovary. The *igf-1* gene was reported to be involved in early vitellogenesis and ovary maturation in the sterlet, *Acipenser ruthenus* [41, 42]. It is then suspected that *igf-1* expression occurred later in the vitellogenesis stage in the Chinese sturgeon.

The testicular tissue was in the early spermatogenesis stage with many primary spermatocytes and a few of spermatogonia (Figure A in S1 Fig). It is thus indicated that some spermatogonia differentiated into primary spermatocytes through meiosis, while a small group of spermatogonia maintained the undifferentiated state. In mice, nanos homolog 3 (*nanos3*) is responsible for maintaining the undifferentiated state of spermatogonia by the control of their cell cycle [43]. Other markers of undifferentiated spermatogonia such as *oct4* (also known as *pou2*), *neurogenin3* and SRY (sex determining region Y)-box 3 (*sox3*) have also been identified, but with no specific function [44–46]. In the Chinese sturgeon transcriptome, *nanos3b* and the undifferentiated spermatogonia markers *sox3* and *oct4* were present, both with higher expression levels in the ovary compared to that in the testis (S4 Table). Our previous study revealed that *oct4* (*pou2*) was transcribed in gonads of immature Chinese sturgeons, including the testes of 1-year-old males that was primarily composed [30], indicating its possible role in the early development of spermatogonia. Besides, the meiosis specific markers of DNA meiotic recombinase 1 (*dmc1*) and meiotic protein covalently bound to DSB (*spo11*) [12] were also found in the present transcriptome. The anti-mullerian hormone (*amh*) gene was another candidate factor involved in testis differentiation, with up-regulated mRNA expression in the testis in some teleosts including zebrafish [47], Nile tilapia [48], rainbow trout [49], sea bass [50] and Japanese flounder [51]. In this study, *amh* was also present in the Chinese sturgeon transcriptome, with higher transcription level in testis than in ovary (Fig 4 and S4 Table), and its role in testicular differentiation of sturgeons requires future investigation.

For the initial phases of ovarian development, limited genes were reported to be responsible. For instance, no stage specific proteins were discovered in the proteome display of immature follicles of zebrafish and the gilthead seabream [52]. In mammals, two members of TGF-β family, the growth and differentiation factor 9 (*gdf9*) and bone morphogenetic factor 15 (*bmp15*) were proved to be involved in early ovarian follicle growth. In this study, *bmp15* was transcribed with higher levels in the ovary than that in the testis (S4 Table), indicating its potential role in the primary oocyte growth in the Chinese sturgeon. Cytochrome P450 aromatase, encoded by *cyp19a*, represents the crucial enzyme that converts androgens to estrogens in the steroidogenic pathway [53]. In our study, *cyp19a1a* was transcribed only in the ovary of the immature Chinese sturgeon, confirming its role in controlling conversion of estradiol-17β, the main regulator of the ovarian development [17]. The forkhead transcription factor (*foxl2*) was another gene transcribed only in ovary of the Chinese sturgeon (Fig 4 and S4 Table), which is the same with that in mouse embryos, chickens, and turtle [54]. The *foxl2* was found to play an essential role in early ovarian development and sex determination, as well as a later role in granulosa cell differentiation with subsequent follicle depletion, and mutations of FOXL2 conduce to a variety of conditions and disease states [55]. Concerning its significant role in the ovary, it would be necessary to explore the function of *foxl2* further in the Chinese sturgeon.

Among the reported sex-determining genes, transcription factor double sex and mab-3 related transcription factor 1 (*dmrt1*) is the only one conserved both in invertebrates and vertebrates essential for male determination [56, 57]. The *dmrt1* expression was revealed to be sexually dimorphic in gonads of lake sturgeon [58] and Siberian sturgeon [59, 60], but not in the shovelnose sturgeon [61]. In this study, no significant difference in *dmrt1* expression was observed between the ovary and testis of Chinese sturgeon. It has been reported that obvious sex differentiation of the Chinese sturgeon can be distinguished early in the 9-month-old sturgeons using surgical operation [19, 62]. Therefore, it is reasonable to speculate that the sexual dimorphism of *dmrt1* might occur in the early stages of gonad differentiation. Besides，in teleost fish, *dmrt1* is assumed to be involved in testis differentiation, considering its expression pattern during the early period of gonad development [48, 49] and on its behavior when masculinizing and feminizing treatments are used [59]. In spermatogonia, *dmrt1* was critical for determining whether spermatogonia go through mitosis and spermatogonial differentiation or meiosis [63]. Thus it would be worth the effort to examine the specific role *dmrt1* plays during the early gametogenesis in Chinese sturgeon. Two other DM domain genes *dmrt3* and *dmrt5* were also found in the Chinese sturgeon transcriptome (Fig 4 and S4 Table). The *dmrt3* was reported to function cooperatively with *dmrt1* after gonadal differentiation in *Takifugu rubripes* [64]. In zebrafish, *dmrt5* was expressed in developing brain and germ cells, evidenced their potential involvement in the HPG axis [65].

*sox9*, an important gene expressed during testis development in mammals [66], was found in the gonad transcriptome of Chinese sturgeon. Together with *dmrt1*, *sox9* is a key SRY target in mammalian testis development, but few studies of *sox9* have been conducted in fish [20]. A study of *sox9* in the Siberian sturgeon revealed it to play a role in late testis differentiation, but not to be a key factor throughout the development of male gonad [60]. Similarly to the expression of *dmrt1*, that of *sox9* in the Chinese sturgeon did not differ significantly in ovary and testis. Further studies were needed to investigate their roles in early gonad differentiation in younger fish. The sex-determination gene *gsdf* was also detected in the Chinese sturgeon transcriptome, again with no significant expression difference between the testis and ovary. Whether the *gsdf* gene, as shown to be sex-determining in *Oryzias luzonensis* [67], plays the same role in the Chinese sturgeon needs further exploration. Furthermore, recombinant Gsdf promotes the spermatogonia proliferation in rainbow trout [68] and *gsdf* gene expression is relevant to early testicular differentiation in medaka [69]. These results strongly indicate a potential function of *gsdf* in the early gametogenesis in Chinese sturgeon.

In *C*. *elegans*, *fem-1* is part of the signal transduction pathway responsible for sex determination [70], and normal masculinization of somatic and germline tissue [71]. The orthologs of *fem-1* in *C*. *elegans* (*fem1a*) were also identified in mouse and human [72, 73]. In addition, transcriptome sequencing in *Pinctada margaritifera* and *Macrobrachium nipponense* indicated the *fem1a* gene to be a sex determination candidate gene [74, 75]. In the Chinese sturgeon transcriptome, no differential expression of *fem1a* was found between testis and ovary, and its function remains unclear in this species.

In the transcriptome annotation, 13 other genes involved in gametogenesis were found (*ar*, *atrx*, *emx2*, *gata-4*, *fgfr2*, *fhl3*, *lhx1*, *sox4*, *sox11*, *sox17* and *wt1*) (Fig 4 and S4 Table). As their specific functions have not been studied in the Chinese sturgeon yet, they are recommended for additional investigations in the future. Of the 22 genes reported in the Adriatic sturgeon transcriptome [4], 8 (*dax1*, *sox1*, *sox6*, *sox14*, *rspo*, *sf-1*, *fgf9*, and *lhx9*) were not observed in the Chinese sturgeon transcriptome. A transcriptome screen for sex differentiation genes in the lake sturgeon identified 12 (*sox2*, *sox4*, *sox17*, *sox21*, *sox9*, *dmrt1*, *rspo-1*, *wt1*, *wnt4*, *foxl2*, *tra-1*, *fem1*) [58], of which five (*sox2*, *sox21*, *rspo-1*, *wnt4* and *tra-1*) were not detected in our study. These differences could be caused by differences in transcriptome sequencing methods and the species used, as well as by the diversity of the developmental stages of the gonads analyzed. Furthermore, only 23.96% of transcript sequences and 15.33% of unigene sequences presented complete ORFs, which suggested that traditional cDNA library and Sanger’s sequencing method were needed for complete transcriptome sequencing. In other words, not all genes transcribed in the gonad transcriptome were annotated. This might be an additional reason why these genes whose function is related to gonad development were not found in the Chinese sturgeon transcriptome.

The detected genes were primarily up-regulated in the ovary compared with the testis. This is similar to the transcriptome of *Sebastiscus marmoratus*, in which differentially expressed genes were predominantly present in the female [76]. The acellular envelope of the developing oocyte composed of 2–4 isoforms of ZP proteins [77]. In our previous study, three *zp3* genes were characterized in *A*. *sinensis* [78]. In this study, more types of *zp* genes were identified that were the most significantly differentially expressed unigenes in ovary (S5 Table), which is consistent with their essential role in the protection of the oocyte. The function of other differentially expressed unigenes is a topic for further research. The specific relative-fold expression of the differentially expressed genes in ovary and testis were different in the RNA-seq data from that obtained by real-time PCR. It may be that biological replicates, which were more valuable and accurate than was increasing the sequencing depth for detecting differently expressed genes, were not included in the transcriptome sequencing [79].

Chromosome numbers in sturgeon species are variable [1, 80]. *Acipenser sinensis* is believed to be a functional tetraploid [80]. Compared to the diploid *D*. *rerio*, *A*. *sinensis* tends to have a greater number of paralogs in the superfamilies of *sox*, *apolipoprotein* and *cyclin* (Table 4), possibly the result of genome polyploidization. Generally, genes of the *sox* family share conserved domains including the High Mobility Group Box; therefore the unigenes we obtained by BLASTX against the NCBI nr database could show multiple matches with *sox* genes with higher scores. As a result, the full-length cDNA transcripts of these unigenes should be cloned for validation in further studies. In addition, the detailed transcriptome investigation of the functional tetraploid Chinese sturgeon in this study is important for evaluating the functional reduction of ploidy when the genome is sequenced or when the transcriptome of functional diploid or octaploid sturgeon species is available [4].

SSRs are tandem repeat DNA sequences that constitute an important part of eukaryote genomes. Being highly polymorphic, they are increasingly used as marker systems in molecular genetics studies, including research involving genetic diversity assessment, comparative genomics, gene flow characterization, and genetic linkage mapping [81]. For the conservation of the Chinese sturgeon, SSRs can serve as effective genetic markers for quantifying genetic diversity within and among populations of this endangered species. In this study, a set of SSRs was identified in which 65.39% were mononucleotide repeat motifs that might be caused by sequencing. Therefore, only the di-, tri-, tetra-, and penta-repeat motifs (Table 6) found would be suitable for polymorphic microsatellite loci identification. In *A*. *dabryanus*, a set of polymorphic microsatellite loci with the di-nucleotide repeat motif were identified [82]. Following the mono-nucleotide repeat motif, the di-nucleotide repeat motif accounted for the second largest group in the *A*. *sinensis* transcriptome (18.71%), suggesting the existence of diverse di-nucleotide repeat motif loci in *A*. *sinensis* similar to those of *A*. *dabryanus*. SSRs identified from the unigenes are useful for description of genealogy and assessment of genetic diversity. The dataset will make contributions to the identification of the molecular mechanism controlling sexual dimorphism and sexual development regulation of *A*. *sinensis*, as well as to the better conservation of this endangered species.

## Supporting Information

S1 Fig Structural characteristics of testicular (A) and ovarian tissue (B) for immature 3-year-old Chinese sturgeons detected by HE staining. Labels: O, pre-vitellogenic oocyte; PSC, primary spermatocyte; SG, spermatogonia.
(TIF)

S1 Table ORF prediction of transcript sequences using TransDecoder. Sequence Headers containing "type:complete" represent transcript sequences with complete ORFs.
(TXT)

S2 Table ORF prediction from unigene sequences using TransDecoder. Sequence Headers containing "type:complete" represent unigene sequences with complete ORFs.
(TXT)

S3 Table KEGG pathways found in the *A*. *sinensis* transcriptome. (XLSX)

S4 Table Genes with potential function in gametogenesis in *A*. *sinensis* transcriptome. (XLSX)

S5 Table List of differentially expressed unigenes in testis compared to ovary. (XLSX)

S6 Table Primers for relative real-time PCR. (DOCX)
