# Supplementary figures and images for: Sequencing and De Novo Assembly of the Gonadal Transcriptome of the Endangered Chinese Sturgeon (Acipenser sinensis)
Source: PLoS One. 2015 Jun 1;10(6):e0127332. doi: 10.1371/journal.pone.0127332 (PMC4452307; doi:10.1371/journal.pone.0127332)

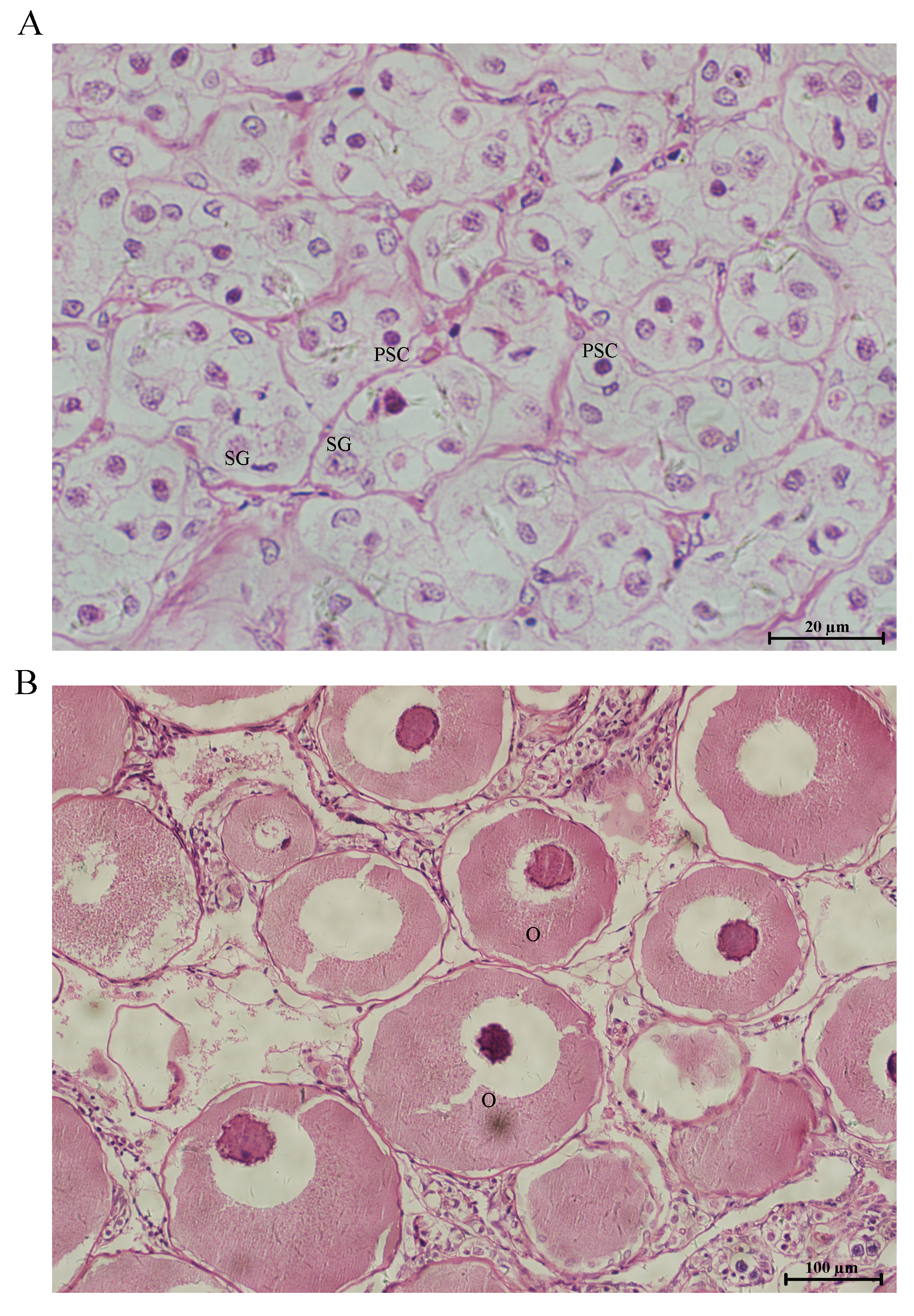

Supplement: S1 Fig — Labels: O, pre-vitellogenic oocyte; PSC, primary spermatocyte; SG, spermatogonia. (TIF) [file pone.0127332.s001.tif]
