# Supplementary material for: Sequencing and De Novo Assembly of the Gonadal Transcriptome of the Endangered Chinese Sturgeon (Acipenser sinensis)
Source: PLoS One. 2015 Jun 1;10(6):e0127332. doi: 10.1371/journal.pone.0127332 (PMC4452307; doi:10.1371/journal.pone.0127332)
Supplement: S6 Table — (DOCX) [file pone.0127332.s007.docx]

**Table S6 Primers for real-time PCR**

|  |  |
| --- | --- |
| ID of Primer | Sequence of primer (5'-3') |
| GnRH2 | TTTGTGGTGATGGGGATGTTGATG |
|  | AGGGCGTCCAGCAGTATTGTCTTC |
| Kiss1 | ATGAATCTCCTGAAGAAACATCACTCCG  GAGACCACCACTGTGTGTTTCGCTGGGGAT |
| Kiss2 | GAGCCCGTTCCAGACTCCAAGCAGCACTAT  GCTCAGGTAAAGCATCATTGGCAGCAGGTG |
| ERalpha | ATGATGAAAGGAGGTATTCGTAAGG  CTCGGTGTATGGGCGGCTGTGTTTC |
| ERbeta1 | ACGGTGTCTGGTCGTGTGAAGGGTGC  GTTCCTTGTCAGCGAGGTTGGTTAG |
| ERbeta2 | AGGTGGTTAGCATAGCGTTGAGCCC  CTTTGTCAGCCAGGTTGGTCAGTGAC |
| Vtgb1 | TTATGGCGAGAGGAAAGATCCGTAC  GCATAGGTGGTGACAGTAATCGGCA |
| Vtg b2 | GAAATCAAGGGATGTGTGGAGGTGC  CAGGACACCATCAGCACACAACTTC |
| AR | ACTTGAGTTCAGAAGGTACGGCAGC  GCCTTTTATCTGTAACCCCTACGAG |
|  |  |
|  |  |
| *β-actin*-F |  |
| *β-actin*-R |  |
